# Evaluation of the consistency of the MRI- based AI segmentation cartilage model using the natural tibial plateau cartilage

**DOI:** 10.1186/s13018-024-04680-5

**Published:** 2024-04-17

**Authors:** Changjiao Sun, Hong Gao, Sha Wu, Qian Lu, Yakui Wang, Xu Cai

**Affiliations:** 1 Joint Diseases Center, Beijing Tsinghua Changgung Hospital, School of Clinical Medicine, Tsinghua University, No. 168 Litang Road, Dongxiaokou Town, Changping District, Beijing, 102218 China; 2 Beijing MEDERA Medical Group, Beijing, 102200 China; 3 Nuctech Company Limited, Beijing, 100083 China; 4 Radiology Department, Beijing Tsinghua Changgung Hospital, School of Clinical Medicine, Tsinghua University, Beijing, China

**Keywords:** Artificial intelligence, Knee osteoarthritis, Articular cartilage segmentation, Magnetic resonance imaging

## Abstract

**Objective:**

The study aims to evaluate the accuracy of an MRI-based artificial intelligence (AI) segmentation cartilage model by comparing it to the natural tibial plateau cartilage.

**Methods:**

This study included 33 patients (41 knees) with severe knee osteoarthritis scheduled to undergo total knee arthroplasty (TKA). All patients had a thin-section MRI before TKA. Our study is mainly divided into two parts: (i) In order to evaluate the MRI-based AI segmentation cartilage model’s 2D accuracy, the natural tibial plateau was used as gold standard. The MRI-based AI segmentation cartilage model and the natural tibial plateau were represented in binary visualization (black and white) simulated photographed images by the application of Simulation Photography Technology. Both simulated photographed images were compared to evaluate the 2D Dice similarity coefficients (DSC). (ii) In order to evaluate the MRI-based AI segmentation cartilage model’s 3D accuracy. Hand-crafted cartilage model based on knee CT was established. We used these hand-crafted CT-based knee cartilage model as gold standard to evaluate 2D and 3D consistency of between the MRI-based AI segmentation cartilage model and hand-crafted CT-based cartilage model. 3D registration technology was used for both models. Correlations between the MRI-based AI knee cartilage model and CT-based knee cartilage model were also assessed with the Pearson correlation coefficient.

**Results:**

The AI segmentation cartilage model produced reasonably high two-dimensional DSC. The average 2D DSC between MRI-based AI cartilage model and the tibial plateau cartilage is 0.83. The average 2D DSC between the AI segmentation cartilage model and the CT-based cartilage model is 0.82. As for 3D consistency, the average 3D DSC between MRI-based AI cartilage model and CT-based cartilage model is 0.52. However, the quantification of cartilage segmentation with the AI and CT-based models showed excellent correlation (r = 0.725; *P* values < 0.05).

**Conclusion:**

Our study demonstrated that our MRI-based AI cartilage model can reliably extract morphologic features such as cartilage shape and defect location of the tibial plateau cartilage. This approach could potentially benefit clinical practices such as diagnosing osteoarthritis. However, in terms of cartilage thickness and three-dimensional accuracy, MRI-based AI cartilage model underestimate the actual cartilage volume. The previous AI verification methods may not be completely accurate and should be verified with natural cartilage images. Combining multiple verification methods will improve the accuracy of the AI model.

## Introduction

Knee osteoarthritis (KOA) is a prevalent type of degenerative joint disease with multiple contributing factors, potentially resulting in functional impairment [1]. Early detection and accurate measurement of cartilage morphology and composition changes are crucial for effectively treating KOA [2]. Magnetic resonance imaging (MRI) provides enhanced visualization of cartilage morphology, making it the preferred modality for assessing cartilage damage and accurately detecting morphological changes [3]. Numerous observational and interventional studies [4–6] have used semi-quantitative and quantitative evaluations of cartilage morphology as an outcome measure because it is sensitive to OA-related change and therapy interventions [7]. Semi-quantitative morphometric assessments are carried out using scoring systems like the Knee Osteoarthritis Scoring Systems (KOSS) [8], the Boston Leeds Osteoarthritis Knee Score (BLOKS) [9], MRI Osteoarthritis Knee Score (MOAKS) [10], and the Whole-Organ MRI Score (WORMS) [11]. Quantitative morphometric techniques utilize three-dimensional MRI data to measure parameters of cartilage tissue, such as cartilage volume, cartilage surface area, total subchondral bone area, and cartilage thickness as biomarkers of osteoarthritis severity and progression [12].

Segmenting the articular cartilage is necessary for cartilage morphometry to compute quantitative parameters. To obtain the quantitative measures, a trained observer must segment the normal cartilage surface and the extent of cartilage damage. This segmentation process is assisted by specialized image analysis software, which is used to calculate the articular cartilage morphometric parameters [12]. However, regardless of the expertise of the trained readers or the capabilities of the segmentation software, the manual or semi-automatic segmentation technique is complex and time-consuming [12].

Recently, deep learning (DL) models of artificial intelligence (AI), specifically convolutional neural networks (CNNs), have emerged as a novel approach for knee cartilage segmentation and KOA diagnosis [13]. Unlike traditional hand-crafted strategies, DL models automatically learn features through multiple layers and numerous parameters [14]. Prasoon et al. [15] developed three 2D CNNs, known as multi-planar CNNs. Their study demonstrated that the learned features from CNNs outperformed hand-crafted features, achieving a volume overlap of 82.49% in DSC. Norman et al. [16] utilized the 2D U-Net model to segment various knee sub-compartments, such as articular cartilage and meniscus. The researchers achieved a mean validation DSC of 86.7% on the OAI dataset, which consisted of 37 subjects. Similarly, Liu et al. [17] employed the 2D SegNet model [18] to segment bone and cartilage in the SKI10 dataset. They also compared the performance of SegNet with U-Net and found that SegNet exhibited superior accuracy and computational efficiency in segmenting musculoskeletal images. In a study by Zhou et al. [19], the CNN model was integrated with a conditional random field with spatial proximity as a supplementary post-processing technique to refine the labeling process. The researchers observed that all cartilages achieved a DSC of over 80%. Ambellan et al. [20] embedded SSMs adjustment into 2D and 3D CNNs as a post-processing step. The authors reported the best results of all published work on the validation data of the SKI10 dataset, which achieved up to 88.3% of DSC on the OAI validation set.

We offered a deep learning-based, fully automatic method for morphological assessment of knee cartilage. It is a new artificial intelligence (AI) segmentation cartilage model based on knee joint thin-section MRI data, which can automatically calculate the cartilage [21]. We all know that the accuracy of AI segmentation is verified by deep learning developers during the AI system development process. AI learning is based on the recognition of imaging data by doctors, and the process of learning imaging data will be affected by the subjectivity of doctors. Therefore, the accuracy of AI recognition may be affected. Over the past years, many studies also have found that there is a certain error in predicting the extent of Osteochondral lesions with MRI [22–24]. If machine learning relies on flawed imaging as the benchmark for accuracy, it will inevitably impact the precision of the machine learning outcomes. One possible approach to tackle these challenges is to employ a pre-trained CNN trained on natural images or diverse medical image modalities and fine-tune it using medical images [25]. In order to avoid this subjective influence and better evaluate and improve the accuracy of our AI segmentation model, our study used the natural tibial plateau cartilage resected during total knee arthroplasty (TKA) as the gold standard for the first time.

The two main objectives of this study were as follows: (i) To evaluate MRI-based AI cartilage's two-dimensional (2D) using the natural tibial plateau as gold standard (ii) To evaluate MRI-based AI cartilage's 2D and three-dimensional (3D) accuracy using the CT-based cartilage model as gold standard.

## Material and methods

### Study population and dataset

This retrospective study was approved by the institutional board of Beijing Tsinghua Changgung Hospital (No. 22383-4-02). The authors maintained complete control over all patient information and imaging data throughout the study. Between May 2021 and May 2022, 33 patients were collected (41 participant knee images in 41 volumes) from Beijing Tsinghua Changgung Hospital, School of Clinical Medicine, Tsinghua University.

The inclusion criteria were: (i) all patients were diagnosed with primary KOA before the operation; (ii) all patients had a knee thin-section MRI scan (PhilipsIngeniaCX, 3.0t) before the operation; (iii) the same senior orthopedic surgeon performed all operations, and (iv) the tibial plateau cartilage was obtained in all patients during the operation.

The exclusion criteria were (i) rheumatic immune osteoarthritis, traumatic osteoarthritis, and other types of arthritis; (ii) a severe bone defect of the tibial plateau that could not obtain a complete tibial plateau osteotomy during operation; and (iii) a preoperative knee joint MRI examination could not be completed due to metal implants.

### The process of the study

**Fig. 1:**
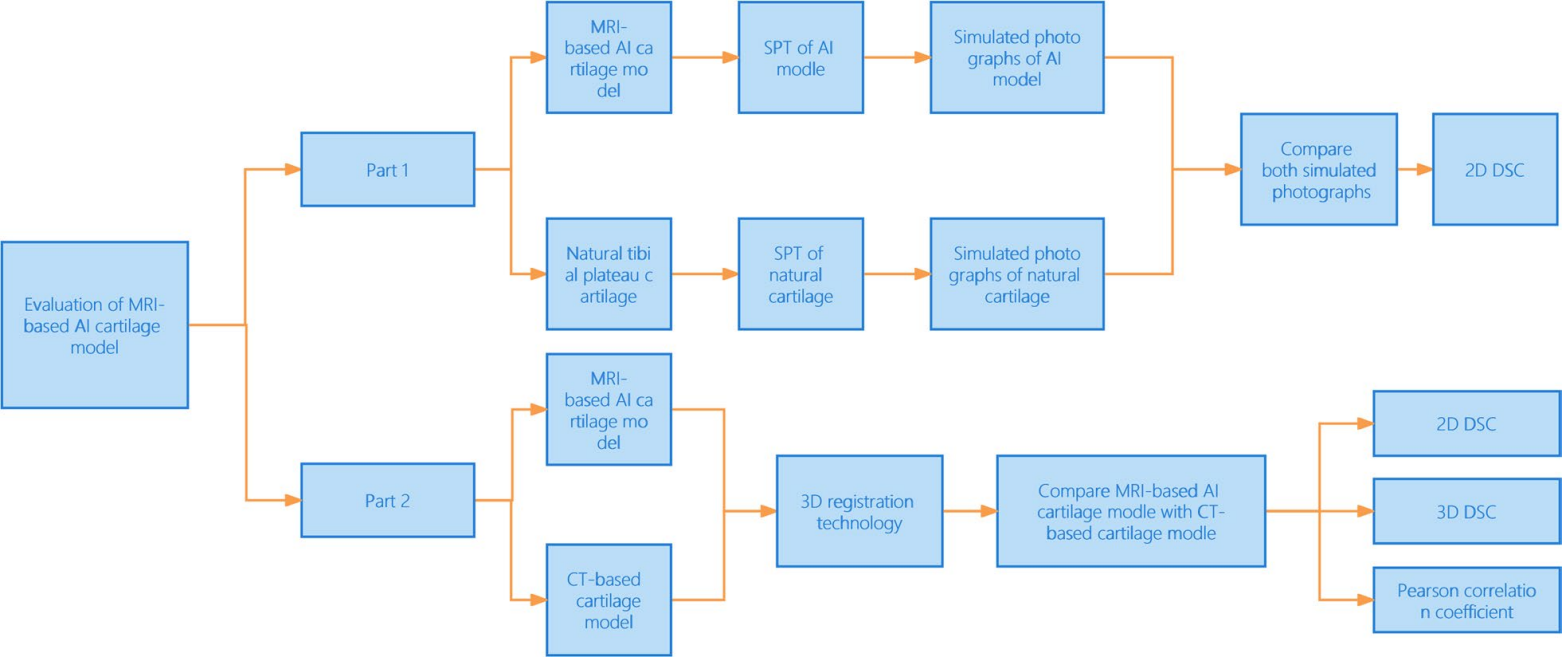
The flow chart of our study process

Our study is mainly divided into two parts: (i) In order to evaluate the MRI-based AI segmentation cartilage model’s 2D accuracy, the natural tibial plateau was used as gold standard. The MRI-based AI segmentation cartilage model and the natural tibial plateau were represented in binary visualization (black and white) simulated photographed images by the application of Simulation Photography Technology (SPT). Both simulated photographed images were compared to evaluate the 2D Dice similarity coefficients (DSC). (ii) In order to evaluate the MRI-based AI segmentation cartilage model’s 3D accuracy. Hand-crafted cartilage model based on knee CT was established. We used these hand-crafted CT-based knee cartilage model as gold standard to evaluate 2D and 3D consistency of between the MRI-based AI segmentation cartilage model and hand-crafted CT-based cartilage model. 3D registration technology was used for both models. Correlations between the AI cartilage model and the CT-based model were also assessed with the Pearson correlation coefficient (PCC). Figure 1 shows the flow chart of our study process.

### Automatic cartilage segmentation

After the patient was admitted to the hospital, a thin-section (slice thickness of 0.7mm) MRI scan of the surgical knee was performed. The MRI of sagittal proton density-weighted fat suppression (PD-FS) sequences were used. All MR scanning was performed on a Philips Ingenia 3T CX MRI (volume size 400 × 400 × 300; voxel size 0.4 × 0.4 × 0.4 mm). The thickness of the segmentation model is 0.4mm, because there is a 0.3mm interval between each layer thickness. The scanned data were imported into an MRI-based intelligent segmentation system [21] developed by our hospital in cooperation with Beijing Tongfang Nuctech Technology Co., Ltd.

**Fig. 2:**
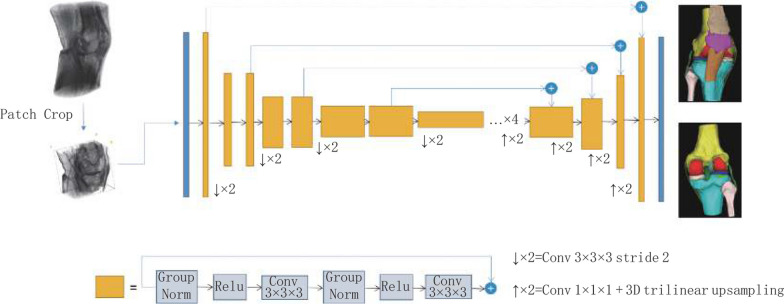
Overview of the SegResNet network architecture. The encoder network down-sample four times, and the residual block amounts of each stage are 1, 2, 2, 2, 4

The AI segmentation system used the convolutional neural network structure of three-dimensional SegResNet to train the segmentation model in a supervised manner using the gradient descent method. An overview of the adopted SegResNet is illustrated in Fig. 2. SegResNet was based on a typical encoder-decoder structure [26] consisting of an encoding sub-network and a corresponding decoding sub-network. In the first stage, the encoder uses 3D convolution with kernels size of (3,3,3) and stride (2,2,2) for down sampling. Down-sampling layer is repeated four times to achieve sufficient data compression, and the last feature map is reduced to 1/16 of the input volume. The initial number of filters is 16, and the number of filters is doubled each time when the feature map is down sampled. Due to the limitation of GPU memory, fixed-size patches are cropped with a random center from the whole volume and then imported to the model. To generate pixel-wise label, the feature map shape is restored to the input volume shape through 3D convolution with kernel size of (1,1,1) and 3D trilinear up-sampling.

**Fig. 3:**
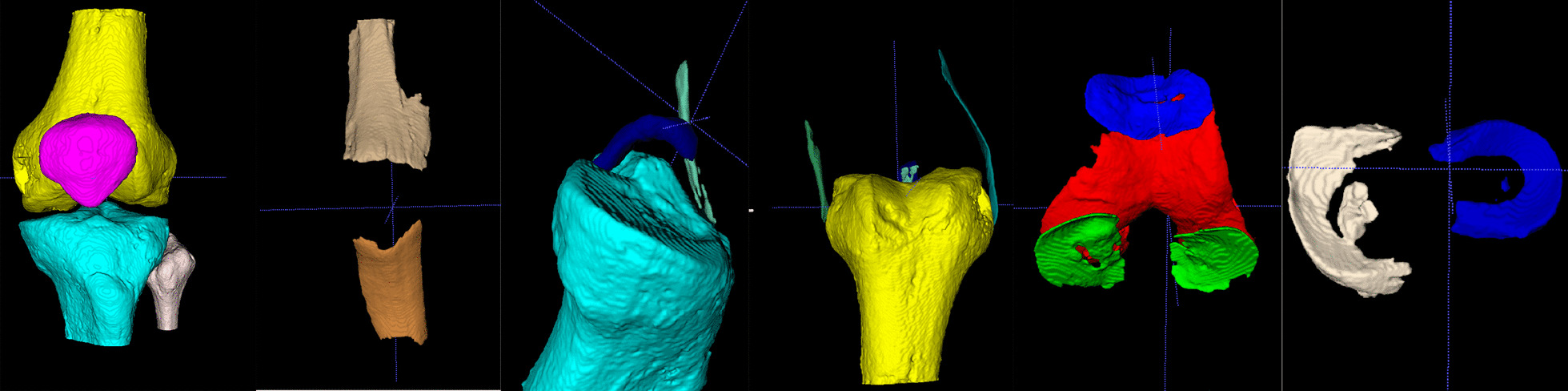
Segmentation results. From left to right are bone, quadriceps tendon and patellar tendon, ACL and PCL, MCL and LCL, cartilage, meniscus

The system can automatically segment knee joint MRI data and quickly obtain bone and important soft tissue segmentation results. The average segmentation accuracy of the system is 92.92% in our previous study [21]. As shown in Fig. 3, this system can segment 15 anatomical structures of the knee accurately, including ﻿four kinds of bones of femur, tibia, patella, and fibula, and three kinds of cartilage, as well as other soft tissue such as meniscus, tendons, anterior cruciate ligament (ACL), posterior cruciate ligament (PCL), medial collateral ligament (MCL) and lateral collateral ligament (LCL).

### SPT of the MRI-based AI model

**Fig. 4:**
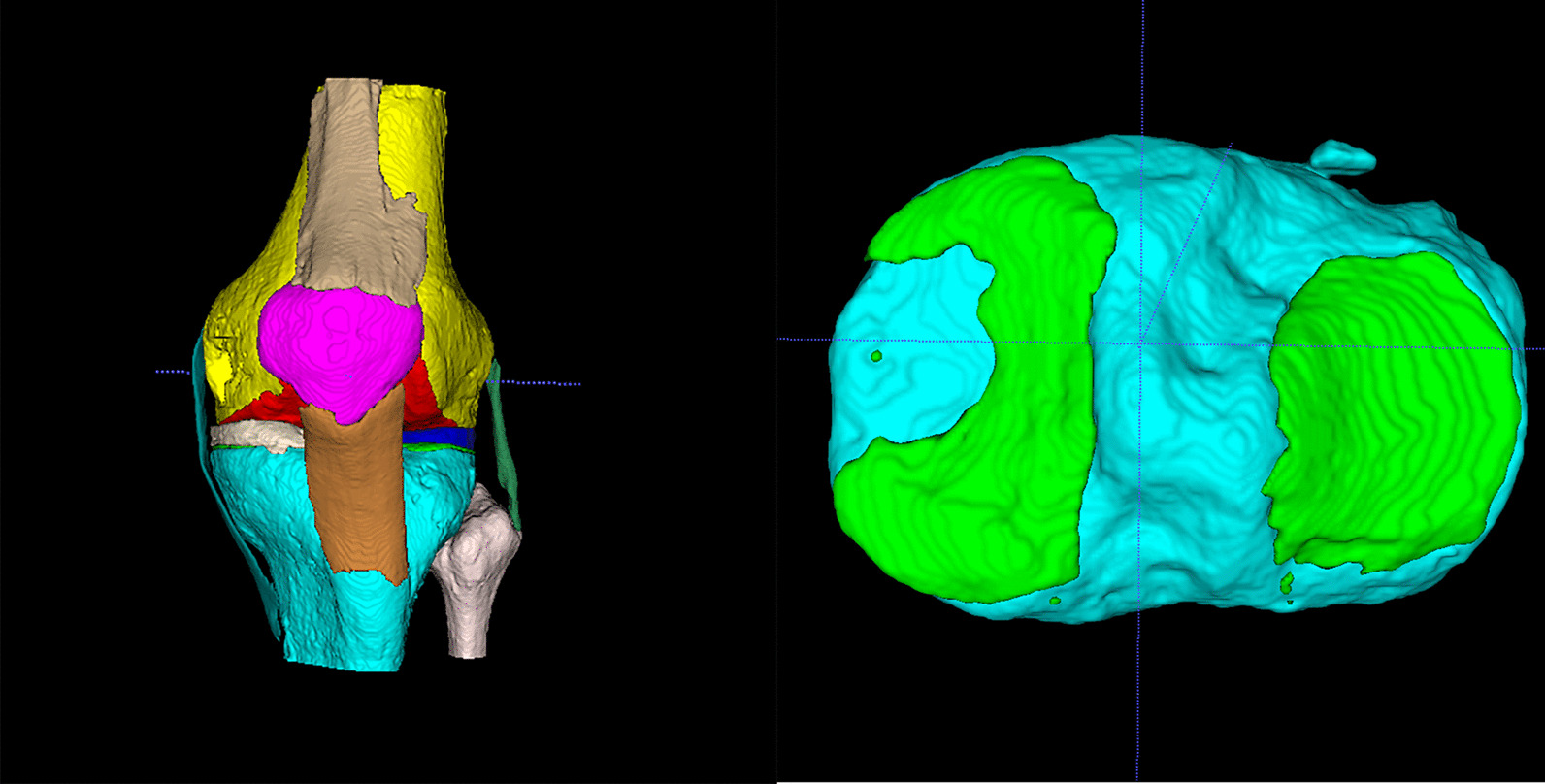
MRI-based intelligent segmentation of knee joint images. **a** MRI-based AI segmentation model; **b** selected tibial image, in which the green part is tibial cartilage

**Fig. 5:**
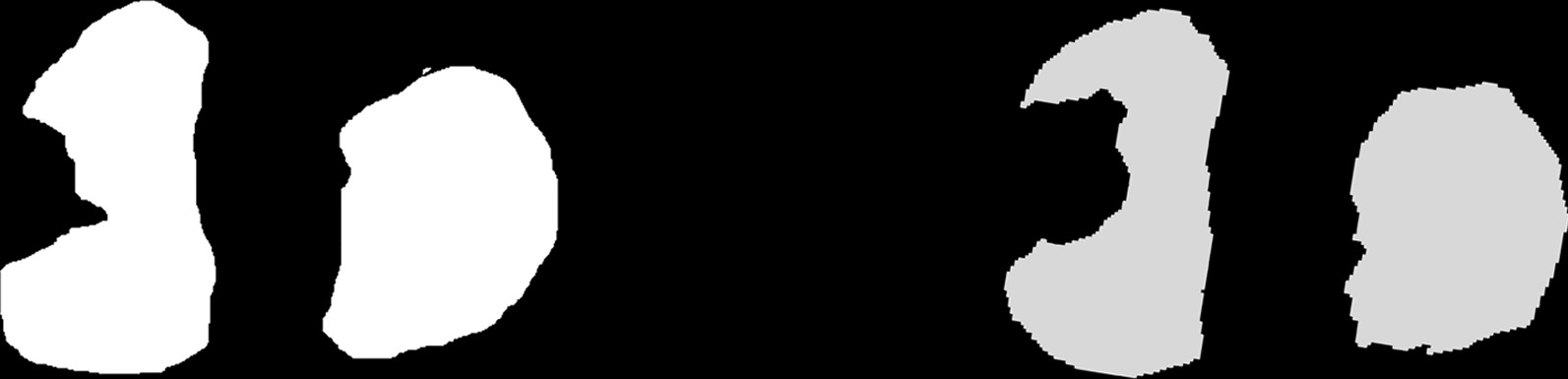
Binary visualization to simulate the photo. From left to right: Natural tibial plateau cartilage, MRI-based AI cartilage model

The tibial plateau cartilage model was included in the test based on the knee model reconstructed by the AI system (Fig. 4). First, Principal Component Analysis (PCA) was employed to rectify the AI MRI-based model. Second, The SPT was used to obtain a simulated two-dimensional image of the AI MRI cartilage model. We restored the same parameters as used when taking pictures of the natural tibial plateau (The camera was 14cm from the plane, the focal length was 1.3 times, the photo background was 1 × 1 mm grid landmark paper). Then, a simulated two-dimensional image of the MRI-based AI cartilage model was obtained. Third, binary visualization (black and white) of the AI MRI-based model’s simulated photographed images was performed to avoid statistical bias as soon as possible (Fig. 5).

### Acquisition of natural tibial plateau cartilage

The same surgeon performed all the operations. The model of the pendulum saw was BJ1101-16659, and the saw blades included 100mm × 22mm and 100mm × 14mm. The saw blades of 100mm × 22mm was used for tibial osteotomy. Most of the ligament stump was removed from the tibial plateau cartilage. The resected tibial plateau was cleaned with normal saline and placed on a mesh paper of 1 × 1mm.

### SPT of the natural tibial plateau cartilage

**Fig. 6:**
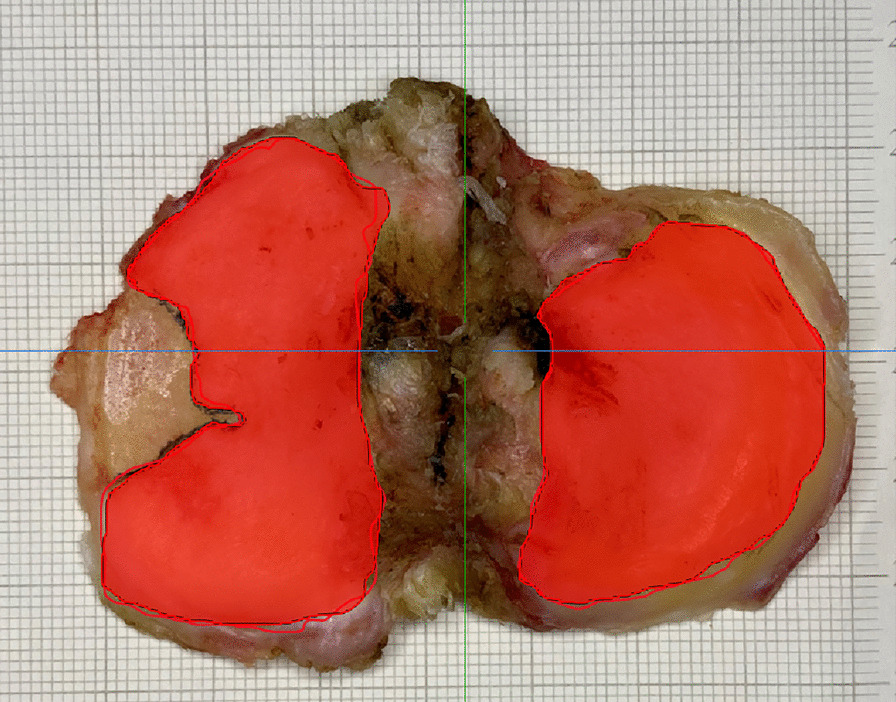
Illustration of the tibial plateau photo taken during the operation. The red part of the tibial plateau was the hand-crafted cartilage

The SPT parameters of the natural tibial plateau cartilage were the same as that of AI MRI-based cartilage model. The camera was vertically fixed above the tibial plateau cartilage to take two-dimensional images of the front face of the tibial plateau cartilage. The camera was 14cm from the plane, and the focal length was 1.3 times. To facilitate the magnification calculation, the photo background as 1 × 1 mm grid landmark paper was chosed (Fig. 6). Binary visualization (black and white) of the natural tibial plateau’s simulated photographed images was also performed to avoid statistical bias as soon as possible (Fig. 5).

### Establishment of CT cartilage model

**Fig. 7:**
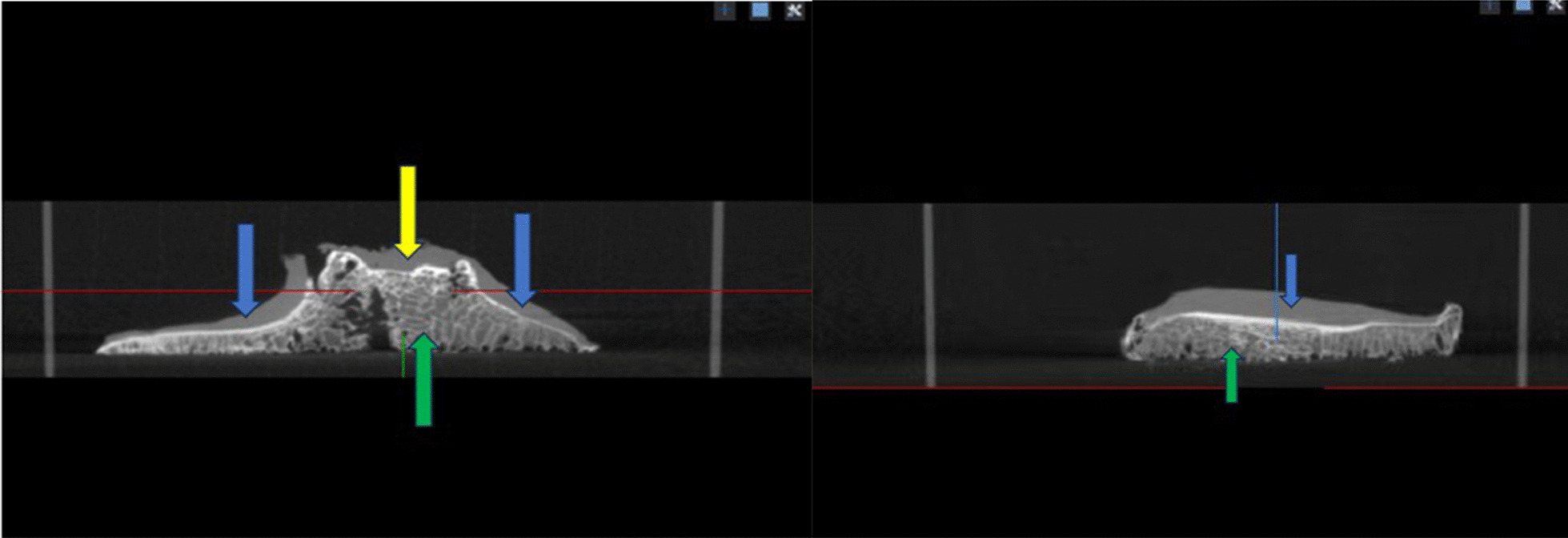
CBCT of the natural tibial plateau cartilage. The picture on the left is the coronal view, and the picture on the right is the sagittal view. Green: bone; blue: cartilage; yellow: residual ligament stumps (soft tissue)

**Fig. 8:**
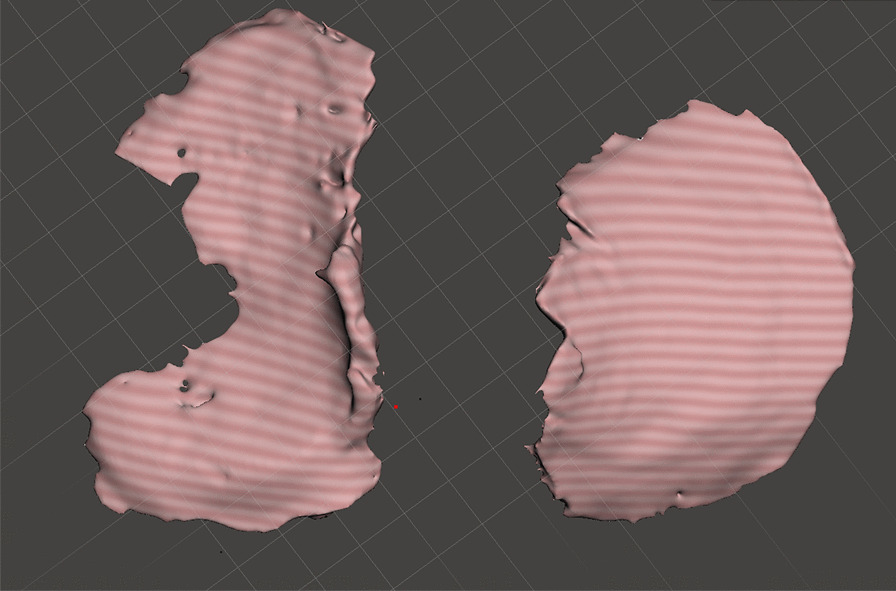
CT cartilage model showing lateral tibial plateau (left) and medial tibial plateau (right)

Although hand-crafted CT cartilage model is time-consuming, it has high consistency and was a gold standard [15]. In order to evaluate the 3D consistency. Hand-crafted cartilage model based on CT was also established. Although Cone Beam Computer Tomography (CBCT) is not as effective as spiral CT in terms of imaging, and CBCT may result in lower spatial resolution and fewer sharp edges, we can obtain the sufficient accuracy we need by using CBCT to carry out our study. In addition, the high cost of spiral CT is another reason why we chose CBCT. A CT scan of the tibial plateau specimen was performed in the Particle Laboratory of Tsinghua University (parameters: CBCT, stadium voltage 120kVp, 160mAs, CT reconstructed image pixel: isotropic 0.2mm) (Fig. 7). The Cartilage was initially labeled with MITK Workbench software. Because SNPA has a small three-dimensional brush that we can use to speed up the annotation of small areas, then the cartilage was accurately labeled through ITK-SNAP software, and finally, a CT cartilage model is established (Fig. 8).

### 3D registration technology

**Fig. 9:**
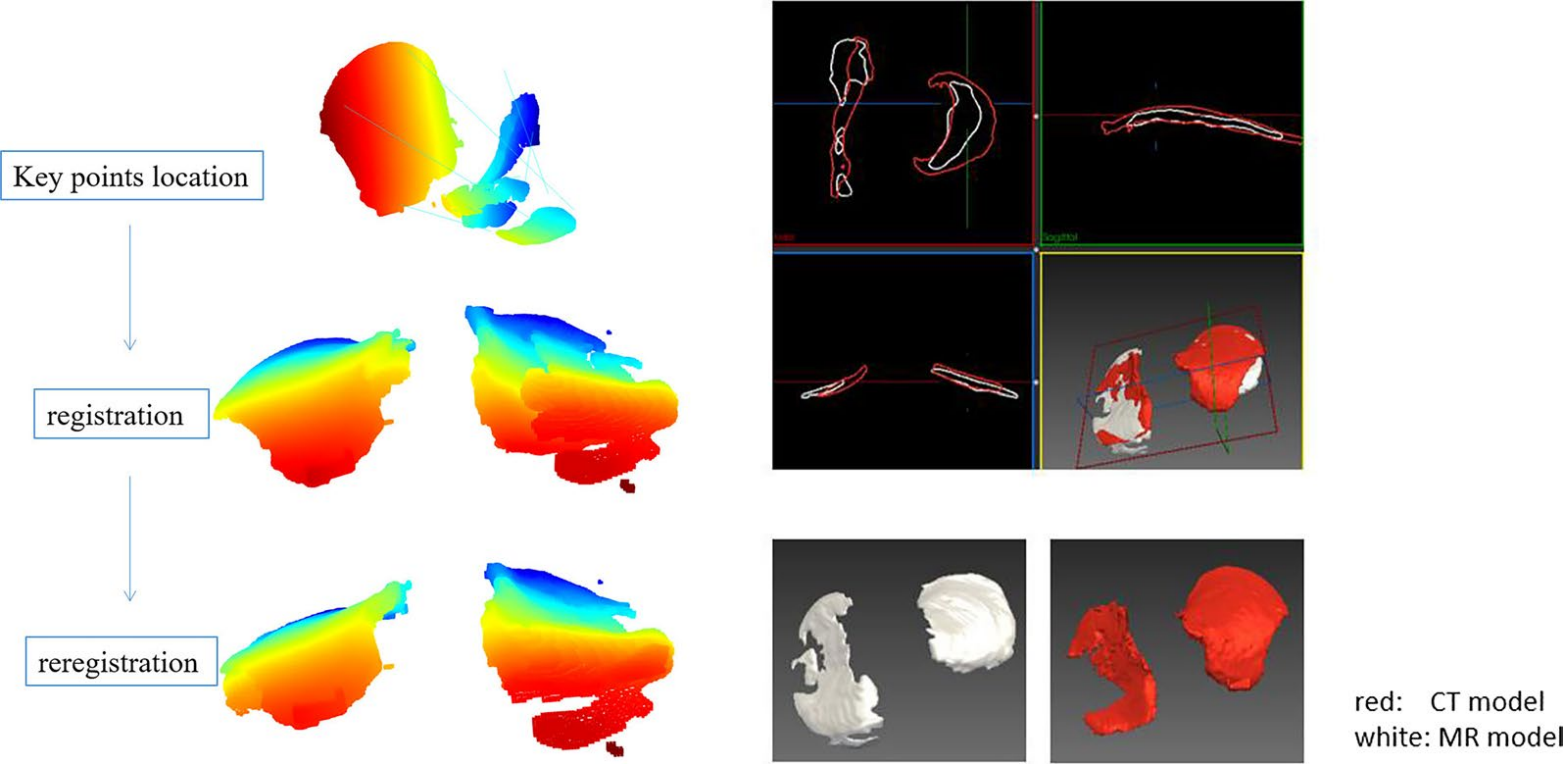
Process of 3D registration technology

When the 3D DSC of the AI cartilage model and the CT cartilage model were verified, we found that the two models could not completely overlap in space, so the DCS could not be calculated directly. So, we did some registration of the two models. A rigid registration method based on the point cloud model was adopted: (i) According to the image characteristics, manually mark some key points (respectively in the corresponding places of the AI reconstructed model and the CT reconstructed model, the front end of the tibia, the medial plateau, the posterior end of the tibia, the outer platform is marked with 6 points in sequence); (ii) Calculate the preliminary registration results based on the key point information; (iii) Due to some objective factors (different image resolutions, different image performances of CT and MR), the key points are not completely accurately correspond to each other, so the ICP (Iterative Closest Point) algorithm is used to adjust the preliminary registration results to make the fine-tuned registration results more accurate (Fig. 9).

### Outcome

The absence of a universally accepted evaluation metric necessitates the utilization of various evaluation metrics to report the segmentation results. An examination of the current body of scholarly work indicates that most of these evaluation metrics are founded on either distance or volume overlap. Among the evaluation measures based on volume overlap, the Dice similarity coefficient (DSC) is the most frequently employed metric [27–31]. Consequently, irrespective of the dataset employed, the DSC serves as this study's primary criterion for comparison. DSC is a quantitative metric used to assess the degree of similarity between two sets of data. It is the quotient of similarity and ranges between 0 and 1 [32]. So DSC was used to evaluate the model segmentation performance.

### Statistical analysis

All statistical analyses were conducted using SPSS27 software (IBM, Armonk, NY, USA). The assessment of normality for continuous variables was conducted using the Kolmogorov–Smirnov test. DSCs were employed to measure the consistency between the AI cartilage model and the natural tibial plateau, as well as between the AI cartilage model and the CT-based model. Correlations between the AI cartilage model and the CT-based model were assessed with the Pearson correlation coefficient (PCC). The Pearson correlation coefficient (PCC) is a correlation coefficient that measures linear correlation between two sets of data. It is the ratio between the covariance of two variables and the product of their standard deviations; thus, it is essentially a normalized measurement of the covariance, such that the result always has a value between − 1 and 1. Statistical significance was defined as 0.05.

## Results

**Fig.10:**
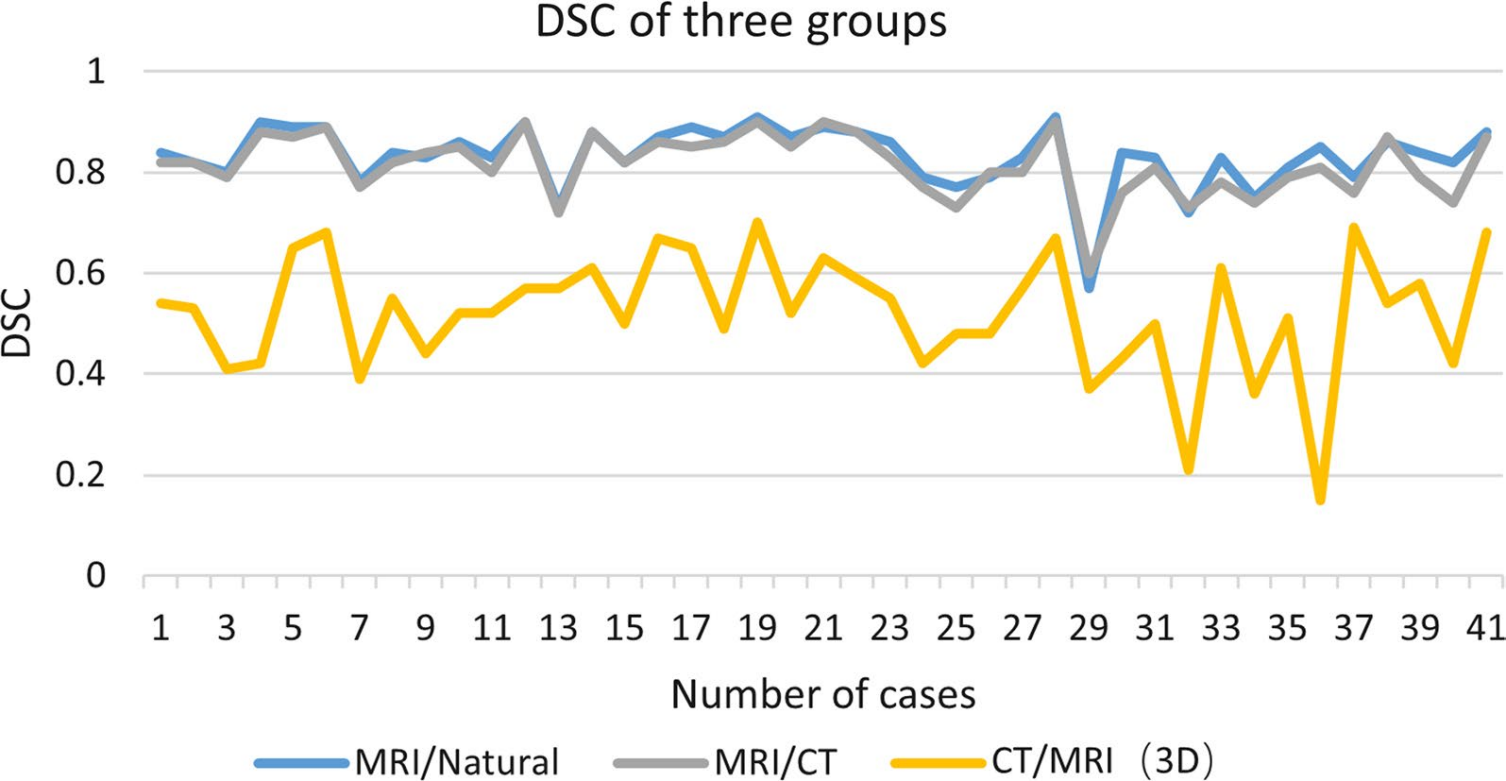
DSC of three groups of results in each case

**Fig. 11:**
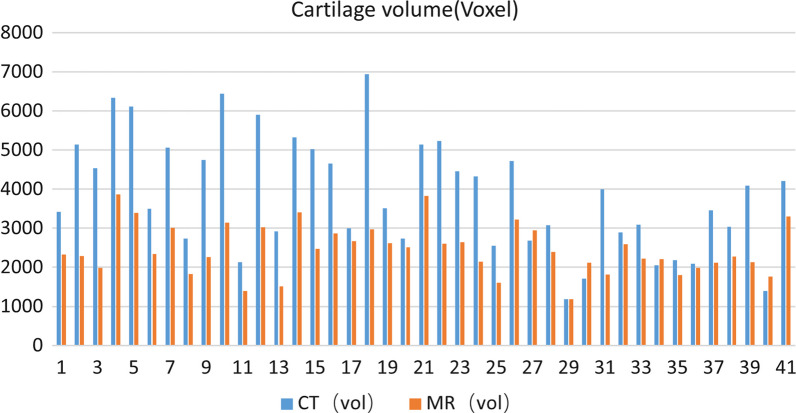
Cartilage volume of each case between AI segmentation cartilage model and the CT-based cartilage model

The AI segmentation cartilage model produced reasonably high 2D DSC. The average 2D DSC between MRI-based AI cartilage model and the tibial plateau cartilage is 0.83. The average 2D DSC between the AI segmentation cartilage model and the CT-based cartilage model is 0.82. As for 3D consistency, the average 3D DSC between MRI-based AI cartilage model and CT-based cartilage model is 0.52. However, the quantification of cartilage segmentation with the AI and CT-based models showed excellent correlation (r = 0.725; *P* values < 0.05). In order to more intuitively show the results of MR-based cartilage segmentation models in each case. Figure 10 visually displayed the 2D and 3D DSC in each case. Figure 11 visually displayed cartilage volume of each case between AI segmentation cartilage model and the CT-based cartilage model.

## Discussion

This is the first study to use the natural tibial plateau cartilage as the gold standard to evaluate the accuracy of the MRI-based AI cartilage segmentation model.

Compared with traditional machine learning that uses MRI as the gold standard, natural tibial plateau cartilage images were used as the gold standard for the first time in our study. We can avoid the subjective influence and better evaluate the accuracy of our AI segmentation model, making the learning performance of the AI model more accurate and objective. This is the main strength of our study.

Most recent investigations on cartilage morphometry use a slice thickness of 1.0 to 1.5 mm, which is within the range recommended by the Osteoarthritis Research Society International (OARSI) clinical trial guidelines [33]. It has been demonstrated that more minor precision errors have been linked to thinner slices [34]. The slice thickness of 0.7mm in our study was another main strength.

In this study, the natural tibial plateau cartilage was employed as the benchmark. The SPT was used to compare the MRI-based AI cartilage segmentation model with the natural tibial plateau cartilage. The mean 2D DSC between the AI reconstructed cartilage model and the natural tibial plateau cartilage was found to be 0.83, and the average 2D DSC between the AI segmentation cartilage model and the CT-based cartilage model is 0.82. which aligns with the findings of previous researches [17–20, 31, 35]. In Zhou’s study [19], they evaluated a new segmentation method using deep convolutional neural network (CNN), 3D fully connected conditional random field (CRF), and 3D simplex deformable modeling to improve the efficiency and accuracy of knee joint tissue segmentation. There were 7 tissue types with mean DSC between 0.8 and 0.9 including the femoral cartilage tibial cartilage, patella, patellar cartilage, meniscus, quadriceps and patellar tendon, and infrapatellar fat pad. In Latif’s study [35], a method for automatic segmentation of the tibiofemoral joint using magnetic resonance imaging (MRI) is presented in their work. The proposed method utilizes a deeply supervised 2D-3D ensemble U-Net, which consists of foreground class oversampling, deep supervision loss branches, and Gaussian weighted softmax score aggregation. The mean DSC of femoral cartilage (90.3 ± 2.89%), and tibial cartilage (86.7 ± 4.07%) is achieved. This study means our MRI-based AI cartilage model can reliably extract morphologic features such as cartilage shape and defect location of the tibial plateau cartilage. This approach could potentially benefit clinical practices such as diagnosing OA.

In order to assess the 3D consistency of the MRI-based AI cartilage model, CT-based cartilage model was also used as gold standard to evaluate 3D consistency of the MRI-based AI cartilage model. The average 3D DSC between MRI-based AI cartilage model and CT-based cartilage model is 0.52. In terms of cartilage thickness and three-dimensional accuracy, MRI-based AI cartilage model underestimate the actual cartilage volume. However, the quantification of cartilage segmentation with the AI cartilage model and the CT-based model also showed excellent correlation. This may mean that our previous AI models based on MRI learning were not so accurate in identifying cartilage thickness. We believe that the reasons for the decline in accuracy of the AI model in predicting cartilage thickness are as follows. First, MRI itself has certain errors in predicting cartilage damage [22–24]. If machine learning relies on these MRI as the benchmark for accuracy, it will inevitably impact the accuracy of the machine learning outcomes. Second, we lacked extensively annotated medical images, which have not yet been fully integrated into clinical practices in our previous AI model learning. Training CNNs from scratch with limited labeled images can result in overfitting [36]. Third, the process of AI learning imaging data will be affected by the subjectivity of doctors. Our results showed that the previous AI verification methods may not be completely accurate and should be verified with real cartilage images. Combining multiple verification methods will improve the accuracy of the AI model.

There are still some limitations in this study. First, the criteria for selecting cases were stringent in terms of inclusion and exclusion. All patients had severe osteoarthritis. There was a lack of comparative data on healthy patients and mild osteoarthritis. Second, Although AI segmentation cartilage model produced reasonably high 2D Dice coefficients. The average 3D DSC between MRI-based AI cartilage model and CT-based cartilage model is not high. MRI-based AI cartilage model underestimate the actual cartilage volume. Thus, additional research with a larger pool of patients on more natural images is necessary to increase cartilage thickness and 3D accuracy of the MRI-based AI cartilage model.

## Conclusion

Our study demonstrated that our MRI-based AI cartilage model can reliably extract morphologic features such as cartilage shape and defect location of the tibial plateau cartilage. This approach could potentially benefit clinical practices such as diagnosing OA. However, in terms of cartilage thickness and three-dimensional accuracy, MRI-based AI cartilage model underestimate the actual cartilage volume. Our results showed that the previous AI verification methods may not be completely accurate and should be verified with real cartilage images. Combining multiple verification methods will improve the accuracy of the AI model. In the future, we will employ the pre-trained CNN trained on more natural images or diverse medical image modalities and fine-tune it to improve cartilage thickness and 3D accuracy of the MRI-based AI cartilage model.

## Data Availability

The datasets produced and/or examined in the present study can be obtained from the corresponding author upon a reasonable request.

## Abbreviations

KOA: Knee Osteoarthritis
OA: Osteoarthritis
MRI: Magnetic resonance imaging
TKA: Total knee arthroplasty
AI: Artificial intelligence
PCA: Principal Component Analysis
SPT: Simulation Photography Technology
PD-FS: Proton density-weighted fat suppression
ACL: Anterior cruciate ligament
PCL: Posterior cruciate ligament
MCL: Medial collateral ligament
LCL: Lateral collateral ligament
DSC: Dice similarity coefficient
TP: True positive
FP: False positive
FN: False negative
KOSS: Knee Osteoarthritis Scoring Systems
BLOKS: Boston Leeds Osteoarthritis Knee Score
MOAKS: MRI Osteoarthritis Knee Score
WORMS: Whole-Organ MRI Score
DL: Deep learning
CNNs: Convolutional neural networks
OARSI: Osteoarthritis Research Society International
CBCT: Cone Beam Computer Tomography

## References

[1] Geng R, Li J, Yu C, Zhang C, Chen F, Chen J (2023). Knee osteoarthritis: current status and research progress in treatment (Review). Exp Ther Med.

[2] Zhang Q, Geng J, Zhang M, Kan T, Wang L, Ai S (2023). Cartilage morphometry and magnetic susceptibility measurement for knee osteoarthritis with automatic cartilage segmentation. Quant Imaging Med Surg.

[3] Omoumi P, Mourad C, Ledoux J-B, Hilbert T (2023). Morphological assessment of cartilage and osteoarthritis in clinical practice and research: intermediate-weighted fat-suppressed sequences and beyond. Skeletal Radiol.

[4] Sabah Afroze AA, Tamilselvi R, Parisa Beham MG (2023). Machine learning based osteoarthritis detection methods in different imaging modalities: a review. Curr Med Imaging.

[5] Butler JJ, Wingo T, Kennedy JG (2023). Presurgical and postsurgical MRI evaluation of osteochondral lesions of the foot and ankle: a primer. Foot Ankle Clin.

[6] Fujiwara H, Yabuuchi H, Wada T, Kobayashi K, Hoshuyama T, Kamitani T (2022). High-resolution magnetic resonance imaging of the triangular fibrocartilage complex using compressed sensing sensitivity encoding (SENSE). Eur J Radiol.

[7] Zibetti MVW, Menon RG, de Moura HL, Zhang X, Kijowski R, Regatte RR (2023). Updates on compositional MRI mapping of the cartilage: emerging techniques and applications. J Magn Reson Imaging.

[8] Kornaat PR, Ceulemans RYT, Kroon HM, Riyazi N, Kloppenburg M, Carter WO (2005). MRI assessment of knee osteoarthritis: knee osteoarthritis scoring system (KOSS)–inter-observer and intra-observer reproducibility of a compartment-based scoring system. Skeletal Radiol.

[9] Hunter DJ, Lo GH, Gale D, Grainger AJ, Guermazi A, Conaghan PG (2008). The reliability of a new scoring system for knee osteoarthritis MRI and the validity of bone marrow lesion assessment: BLOKS (Boston Leeds Osteoarthritis Knee Score). Ann Rheum Dis.

[10] Hunter DJ, Guermazi A, Lo GH, Grainger AJ, Conaghan PG, Boudreau RM (2011). Evolution of semi-quantitative whole joint assessment of knee OA: MOAKS (MRI Osteoarthritis Knee Score). Osteoarthritis Cartilage.

[11] Peterfy CG, Guermazi A, Zaim S, Tirman PF, Miaux Y, White D, , et al. Whole-organ magnetic resonance imaging score (WORMS) of the knee in osteoarthritis. Osteoarthritis and cartilage. 2004 [cited 2023 Oct 21];12. Available from: https://pubmed.ncbi.nlm.nih.gov/14972335/10.1016/j.joca.2003.11.003 14972335

[12] Eck BL, Yang M, Elias JJ, Winalski CS, Altahawi F, Subhas N (2023). Quantitative MRI for evaluation of musculoskeletal disease: cartilage and muscle composition, joint inflammation, and biomechanics in osteoarthritis. Invest Radiol.

[13] Bousson V, Benoist N, Guetat P, Attané G, Salvat C, Perronne L (2023). Application of artificial intelligence to imaging interpretations in the musculoskeletal area: where are we? Where are we going?. Joint Bone Spine.

[14] Ehmig J, Engel G, Lotz J, Lehmann W, Taheri S, Schilling AF (2023). MR-imaging in osteoarthritis: current standard of practice and future outlook. Diagnostics (Basel).

[15] Prasoon A, Petersen K, Igel C, Lauze F, Dam E, Nielsen M (2013). Deep feature learning for knee cartilage segmentation using a triplanar convolutional neural network. Med Image Comput Comput Assist Interv.

[16] Norman B, Pedoia V, Majumdar S (2018). Use of 2D U-net convolutional neural networks for automated cartilage and meniscus segmentation of knee MR imaging data to determine relaxometry and morphometry. Radiology.

[17] Liu F, Zhou Z, Jang H, Samsonov A, Zhao G, Kijowski R (2018). Deep convolutional neural network and 3D deformable approach for tissue segmentation in musculoskeletal magnetic resonance imaging. Magn Reson Med.

[18] SegNet: A Deep Convolutional Encoder-Decoder Architecture for Image Segmentation | IEEE Journals & Magazine | IEEE Xplore. [cited 2023 Oct 21]. Available from: https://ieeexplore.ieee.org/abstract/document/7803544 10.1109/TPAMI.2016.2644615 28060704

[19] Zhou Z, Zhao G, Kijowski R, Liu F (2018). Deep convolutional neural network for segmentation of knee joint anatomy. Magn Reson Med.

[20] Ambellan F, Tack A, Ehlke M, Zachow S (2019). Automated segmentation of knee bone and cartilage combining statistical shape knowledge and convolutional neural networks: Data from the Osteoarthritis Initiative. Med Image Anal.

[21] Le Shen Lu, Qian TH, Sha Wu, Yi Yi, Yunda S (2022). A feasibility study of knee joint semantic segmentation on 3D MR images. Comput Tomogr Theory Appl.

[22] Campbell AB, Knopp MV, Kolovich GP, Wei W, Jia G, Siston RA (2013). Preoperative MRI underestimates articular cartilage defect size compared with findings at arthroscopic knee surgery. Am J Sports Med.

[23] Perry J, Kuiper JH, McCarthy HS, Jermin P, Gallacher PD, Tins B (2023). Comparison of knee articular cartilage defect size between measurements obtained on preoperative MRI versus during arthrotomy. Orthop J Sports Med.

[24] Chen Y, Li Y, Liu W, Wang Z, Li J, Chen C (2023). Comparison of surface microscopy coil and ankle joint special phased array coil magnetic resonance imaging in assessing preoperative osteochondral lesions of the talus. Quant Imaging Med Surg.

[25] Shin H-C, Roth HR, Gao M, Lu L, Xu Z, Nogues I (2016). Deep convolutional neural networks for computer-aided detection: CNN architectures, dataset characteristics and transfer learning. IEEE Trans Med Imaging.

[26] Myronenko A. 3D MRI brain tumor segmentation using autoencoder regularization. Brainlesion: Glioma, Multiple Sclerosis, Stroke and Traumatic Brain Injuries. Springer, Cham; 2019 [cited 2023 Oct 24]. p. 311–20. 10.1007/978-3-030-11726-9_28

[27] Schmidt AM, Desai AD, Watkins LE, Crowder HA, Black MS, Mazzoli V (2023). Generalizability of deep learning segmentation algorithms for automated assessment of cartilage morphology and MRI relaxometry. J Magn Reson Imaging.

[28] Zhang R, Zhou X, Raithel E, Ren C, Zhang P, Li J, et al. A reproducibility study of knee cartilage volume and thickness values derived by fully automatic segmentation based on three-dimensional dual-echo in steady state data from 1.5 and 3 T magnetic resonance imaging. MAGMA. 2023. 10.1007/s10334-023-01122-x 37815638

[29] Kuiper RJA, Colaris JW, Stockmans F, van Es EM, Viergever MA, Seevinck PR, et al. Impact of bone and cartilage segmentation from CT and MRI on both bone forearm osteotomy planning. Int J Comput Assist Radiol Surg. 2023. 10.1007/s11548-023-02929-8 PMC10632286 37219804

[30] Kim H, Shin K, Kim H, Lee E-S, Chung SW, Koh KH (2022). Can deep learning reduce the time and effort required for manual segmentation in 3D segmentation of MRI in rotator cuff tears?. PLoS ONE.

[31] Kulseng CPS, Nainamalai V, Grøvik E, Geitung J-T, Årøen A, Gjesdal K-I (2023). Automatic segmentation of human knee anatomy by a convolutional neural network applying a 3D MRI protocol. BMC Musculoskelet Disord.

[32] Zou KH, Warfield SK, Bharatha A, Tempany CMC, Kaus MR, Haker SJ (2004). Statistical validation of image segmentation quality based on a spatial overlap index. Acad Radiol.

[33] Hunter DJ, Altman RD, Cicuttini F, Crema MD, Duryea J, Eckstein F (2015). OARSI clinical trials recommendations: knee imaging in clinical trials in osteoarthritis. Osteoarthritis Cartilage.

[34] Eckstein F, Charles HC, Buck RJ, Kraus VB, Remmers AE, Hudelmaier M, et al. Accuracy and precision of quantitative assessment of cartilage morphology by magnetic resonance imaging at 3.0T. Arthritis Rheum. 2005;52:3132–6. 10.1002/art.21348 16200592

[35] Latif MHA, Faye I (2021). Automated tibiofemoral joint segmentation based on deeply supervised 2D–3D ensemble U-net: data from the osteoarthritis initiative. Artif Intell Med.

[36] Srivastava N, Hinton G, Krizhevsky A, Sutskever I, Salakhutdinov R (2014). Dropout: a simple way to prevent neural networks from overfitting. J Mach Learn Res.

